# Zebinidae (Caenogastropoda, Rissooidea) from Hainan Island, China, with re-description of four species

**DOI:** 10.3897/BDJ.13.e165463

**Published:** 2025-11-10

**Authors:** Lu Qi, Songyuan Liu, Shengming Sun, Lingfeng Kong, Zhenhua Ma

**Affiliations:** 1 South China Sea Fisheries Research Institute, Chinese Academy of Fishery Sciences, Guangzhou, China South China Sea Fisheries Research Institute, Chinese Academy of Fishery Sciences Guangzhou China; 2 Key Laboratory of Mariculture, Ministry of Education, Ocean University of China, Qingdao, China Key Laboratory of Mariculture, Ministry of Education, Ocean University of China Qingdao China; 3 Key Laboratory of Freshwater Aquatic Resources, Ministry of Agriculture and Rural Affairs, Shanghai Ocean University, Shanghai, China Key Laboratory of Freshwater Aquatic Resources, Ministry of Agriculture and Rural Affairs, Shanghai Ocean University Shanghai China

**Keywords:** Zebinidae, taxonomy, morphology

## Abstract

**Background:**

Hainan Island serves as an important reservoir of molluscan biodiversity in China. However, the documented molluscan fauna is predominantly composed of macromolluscan species, while micromolluscan diversity remains critically understudied.

**New information:**

In this study, we document four marine microgastropod species belonging to the family Zebinidae Coan, 1964 from Hainan Island, China: *Schwartziella triticea* (Pease, 1861), *Pandalosia subfirmata* (O. Boettger, 1887), *Zebina tridentata* (Michaud, 1830) and *Stosicia annulata* (Dunker, 1860). Scanning electron microscopy (SEM) was employed to examine shell morphology. Amongst these, *Stosicia annulata* (Dunker, 1860) represent a new record for China, suggesting that more extensive sampling along Hainan Island's coastline will likely yield additional discoveries.

## Introduction

Hainan Island occupies a strategically important biogeographic position at the northern margin of the South China Sea, representing a critical north-western extension of the Coral Triangle biodiversity hotspot. With a land area of 33,900 km² and a coastline extending 1,535 km, representing approximately 5% of China's total coastline, it serves as a significant reservoir of marine biodiversity. Current documentation records approximately 500 molluscan species (Zhang et al. 2017), predominantly consisting of macromolluscan species, whereas micromolluscan diversity remains critically understudied. However, significant proportions of molluscan diversity are typically represented by smaller-bodied species. A study by Bouchet et al. (2002) demonstrated that, of a total of 2,738 species collected in one site in New Caledonia, almost 52% have an adult size smaller than 8.8 mm. Hainan Island's micromolluscan fauna remains particularly understudied, with preliminary estimates suggesting over 50% of marine mollusc species in this region await discovery because micromolluscs have been rarely sampled and systematically studied. The recent study on Rissoinidae by Qi et al. (2025) and the present contribution on Zebinidae represent initial progress towards comprehensive documentation of marine molluscan biodiversity in Hainan Island.

The family Zebinidae comprises small to minute marine gastropods typically measuring 2-5 mm in adult shell length. Members of this family are predominantly detritivorous, inhabiting tropical and subtropical marine ecosystems worldwide. The family exhibits both planktotrophic and non-planktotrophic larval development strategies (Sleurs and Strack 2021). Current estimates suggest 168 extant species globally (MolluscaBase 2025), with about two-thirds occurring in the Indo-Pacific Region, yet records from the South China Sea remain exceptionally limited. In this study, we document four zebinid species from Hainan Island, China, including one new regional record for Chinese waters.

## Materials and methods

Samples were collected from the intertidal zone of Hainan Island in 2023 and preserved in 95% ethanol. For this study, only shells were examined. To obtain high-resolution shell images, the following preparation protocol was implemented: First, shells were ultrasonically cleaned (Branson 5800, 40 kHz, 10 min) to remove surface debris. Cleaned shells were then mounted on conductive carbon tape and sputter-coated with a 20-nm gold-palladium layer using an ion coater (Hitachi MC1000). Imaging was performed with a scanning electron microscope TESCAN (China) Co., Ltd., Shanghai, China, operated at 15 kV, following established methodologies (Qi et al. 2025). The shells were measured using an eyepiece micrometer fitted on a stereomicroscope. In this study, species identification of micromolluscs was primarily based on external morphological characteristics. Specimens were examined under a stereomicroscope to observe key diagnostic features, including shell morphology and sculpture. These observations were systematically compared with detailed descriptions and illustrations from classical taxonomic monographs, regional faunal guides and relevant taxonomic literature (Okutani 2017, Sleurs and Strack 2021). For specimens with ambiguous identities, cross-referencing across multiple literature sources was conducted. All identifications were verified against authoritative online databases (e.g. WoRMS) to confirm the current validity of scientific names.

## Taxon treatments

### Schwartziella triticea

(Pease, 1861)

A5F87233-2B35-5DEA-A0DE-0F32446B455A

#### Materials

- **Type status:** Other material. **Occurrence:** catalogNumber: XCL59; recordedBy: Qi Lu; individualCount: 1; lifeStage: adult; occurrenceID: 20A6B31E-18D4-513E-9DB0-3DA25563A15D; **Taxon:** taxonID: Native; scientificName: *Schwartziella triticea*; originalNameUsage: *Rissoina triticea* Pease, 1861, *Rissoina rissoi* Weinkauff, 1881; **Location:** country: China; stateProvince: Hainan; locality: Chiling; **Identification:** identifiedBy: Qi Lu; dateIdentified: 2023

#### Description

**Figure 1.**
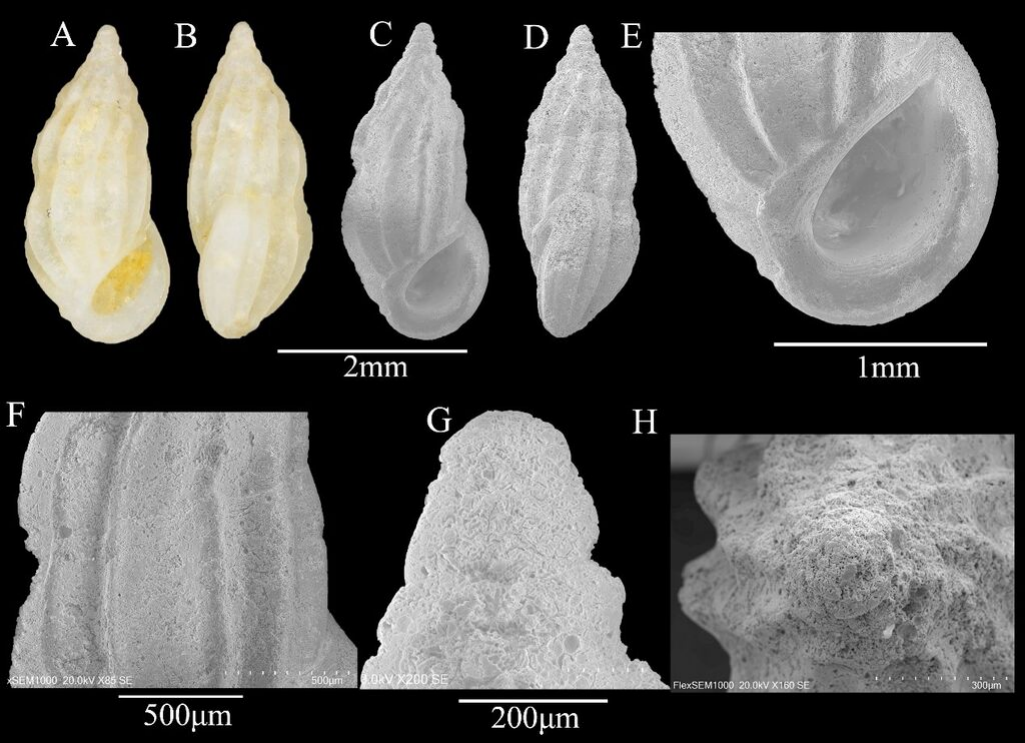
*Schwartziella triticea* (Pease, 1861). **A, B** shell illustration in colour; **C** scanning electron micrographs of frontal view of shell; **D** scanning electron micrographs of lateral view of shell; **E** detail of aperture; **F** detail of teleoconch surface; **G** scanning electron micrographs of lateral view of protoconch; **H** scanning electron micrographs of apical view of protoconch.

Shell minute (3.4 ± 0.25 mm in height; 1.5 ± 0.01 mm in width), fusiform-ovate in shape, white, solid and translucent (Fig. 1A and B). Seven whorls, body whorl height greater than spire (Fig. 1C and D); teleoconch whorls 4, inflated, sculptured exclusively by axial ribs with wide intercostal spaces (approximately twice the width of ribs), axial ribs on body whorl constricted at base (Fig. 1C-F); shell surface with fine microsculpture (Fig. 1F). Suture distinct, but shallow (Fig. 1F). Aperture large, D-shaped, angulated posteriorly, without anterior channel; outer lip prosocline, thick, with varix, bearing fine growth lines on edge; inner lip and columellar lip forming straight line, thick, broad, everted at columella; interior of aperture with delicate annular sculpture (Fig. 1E). Base lacking folds. Umbilicus absent. Protoconch of 3 whorls, eroded (Fig. 1G and H).

#### Diagnosis

Shell somewhat fusiformly ovate; ribs nine, prominent, smooth, continuous over the sutures; aperture ovate (Pease 1861).

#### Distribution

*Schwartziella triticea* exhibits a broad tropical distribution, ranging from the Red Sea across the Indian Ocean. It is also recorded from Hainan Island (China) and Hawaiian waters (Sleurs and Strack 2021).

#### Taxon discussion

The genus *Schwartziella* Nevill, 1881 is primarily characterised by prominent axial ribs on the shell surface, with ribs on the body whorl extending continuously to the base. It differs from *Rissoina* in lacking an anterior canal in the aperture and possessing an inner operculum without peg-like projections. When compared to *Zebina* H. Adams & A. Adams, 1854, *Schwartziella* Nevill, 1881 shows minimal anatomical distinctions, but exhibits more robust axial ribbing. *S. triticea* closely resembles *S. laseroni* (C.-K. Chang & W.-L. Wu, 2004); however, the latter can be distinguished by the presence of basal plicae and a narrower columellar lip.

### Pandalosia subfirmata

(O. Boettger, 1887)

3830653F-2FFB-5DDC-B9C7-B710FDFD9263

#### Materials

- **Type status:** Other material. **Occurrence:** catalogNumber: STGY71; recordedBy: Qi Lu; individualCount: 2; lifeStage: adult; occurrenceID: EDBBA5C5-74AE-557C-B169-350D9E4314E3; **Taxon:** taxonID: Native; scientificName: *Pandalosia subfirmata*; originalNameUsage: *Costalynia decapitata* Laseron, 1956, *Costalynia truncata* Laseron, 1956, *Pandalosia darwinensis* Laseron, 1956, *Pandalosia excelsis* Laseron, 1956, *Pandalosia subulata* Laseron, 1956, Rissoina (Schwartziella) subfirmata O. Boettger, 1887, Schwartziella (Pandalosia) darwinensis (Laseron, 1956); **Location:** country: China; stateProvince: Hainan; locality: Wenchang; **Identification:** identifiedBy: Qi Lu; dateIdentified: 2023

#### Description

**Figure 2.**
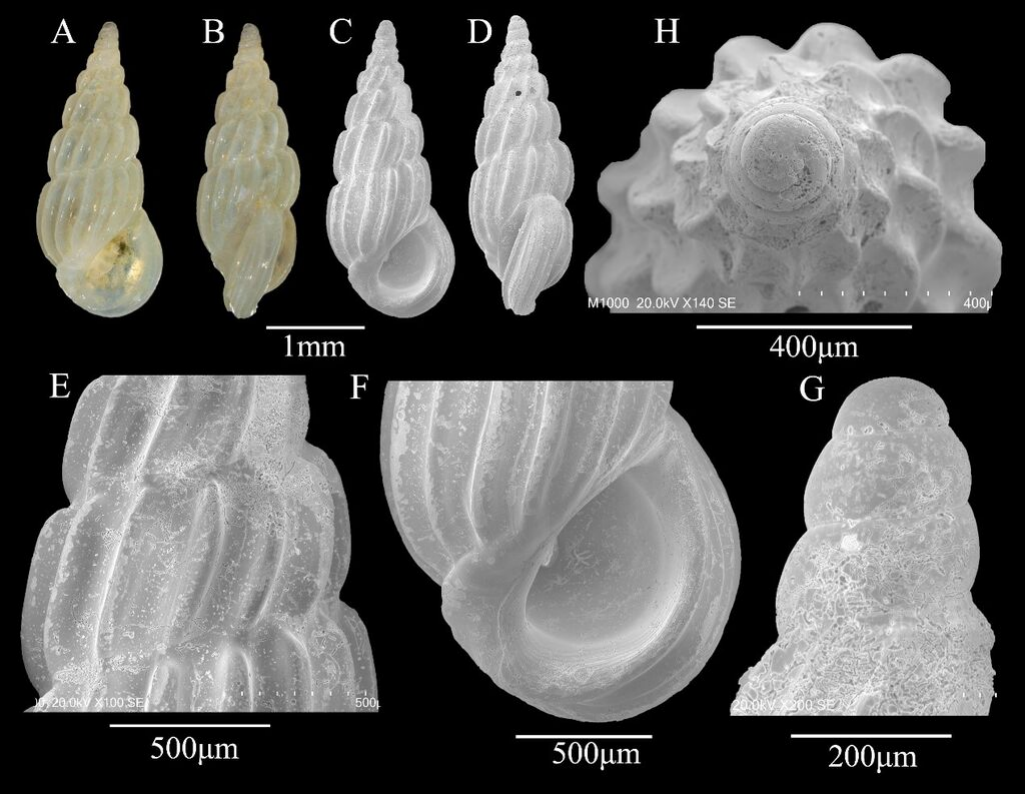
*Pandalosia subfirmata* (O. Boettger, 1887). **A, B** shell illustration in colour; **C** scanning electron micrographs of frontal view of shell; **D** scanning electron micrographs of lateral view of shell; **E** detail of teleoconch surface; **F** detail of aperture; **G** scanning electron micrographs of lateral view of protoconch; **H** scanning electron micrographs of apical view of protoconch.

Shell minute (3 ± 0.15 mm in height, 1 ± 0.02 mm in width), conical, white, glossy, translucent (Fig. 2A-D). Protoconch with 3.5 whorls, conical, slender, smooth, transition to teleoconch poorly defined (Fig. 2G and H). Teleoconch comprising approximately 5 whorls, each convex with deeply impressed sutures; sculpture consisting of prosocline axial ribs, lacking spiral elements; axial ribs sparsely distributed, intercostal spaces exceeding rib width (Fig. 2C-E). Base with prominent plica. Aperture D-shaped, posteriorly angulate, lacking distinct anterior canal; outer lip thickened, varix bearing longitudinal striae; interior of aperture with delicate spiral lirae (Fig. 2F).

#### Diagnosis

Whorls growing fairly rapidly, rather convex, separated by a deep suture; initial whorls smooth, the others strongly ribbed, with rounded, oblique ribs and smooth interspaces. Aperture oblique, narrowly ovate (Boettger 1887).

#### Distribution

Tropical Indo-Pacific Region (China, Japan, Australia); including tropical western America (Sleurs and Strack 2021).

#### Taxon discussion

The genus *Pandalosia* was segregated from *Schwartziella* (Laseron 1956), characterised by smaller and more slender shells; protoconch comparatively large and elongate, surface smooth, lacking microsculpture, base bearing a prominent plica. *Pandalosia subfirmata* (O. Boettger, 1887) is not a new record from Chinese waters, as it had been previously identified in Taiwanese waters as *Pandalosia darwinensis* Laseron,1956 (Liu 2008). Currently, *Pandalosia darwinensis* Laseron, 1956 is treated as a synonym of *P. subfirmata* in the World Register of Marine Species (WoRMS). Two additional species, *Pandalosia moerchiella* C.-K. Chang & W.-L. Wu, 2004 and *Pandalosia lutaoi* C.-K. Chang & W.-L. Wu, 2004, have also been reported from Taiwanese waters, though their taxonomic status remains uncertain. These two species are clearly distinguishable from *P. subfirmata* in shell sculpture: *P. moerchiella* possesses a smooth shell surface lacking prominent axial ribs, while *P. lutaoi* exhibits much weaker and less distinct axial ribs compared to those of *P. subfirmata* (Chang and Wu 2004). A comprehensive taxonomic revision of the genus *Pandalosia* in Chinese waters is warranted in the future.

### Zebina tridentata

(Michaud, 1830)

2392D18F-83D2-53A0-A47C-D041E51EF349

#### Materials

- **Type status:** Other material. **Occurrence:** catalogNumber: XDH61; recordedBy: Qi Lu; individualCount: 3; lifeStage: adult; occurrenceID: 1963B1AA-1DEF-597F-AE68-694D346B6FAF; **Taxon:** taxonID: Native; scientificName: *Zebina tridentata*; originalNameUsage: *Rissoa crassilabrum* Garrett, 1857, *Rissoa tridentata* Michaud, 1830, *Rissoina crassilabrum* (Garrett, 1857); **Location:** country: China; stateProvince: Hainan; locality: Sanya; **Identification:** identifiedBy: Qi Lu; dateIdentified: 2023

#### Description

**Figure 3.**
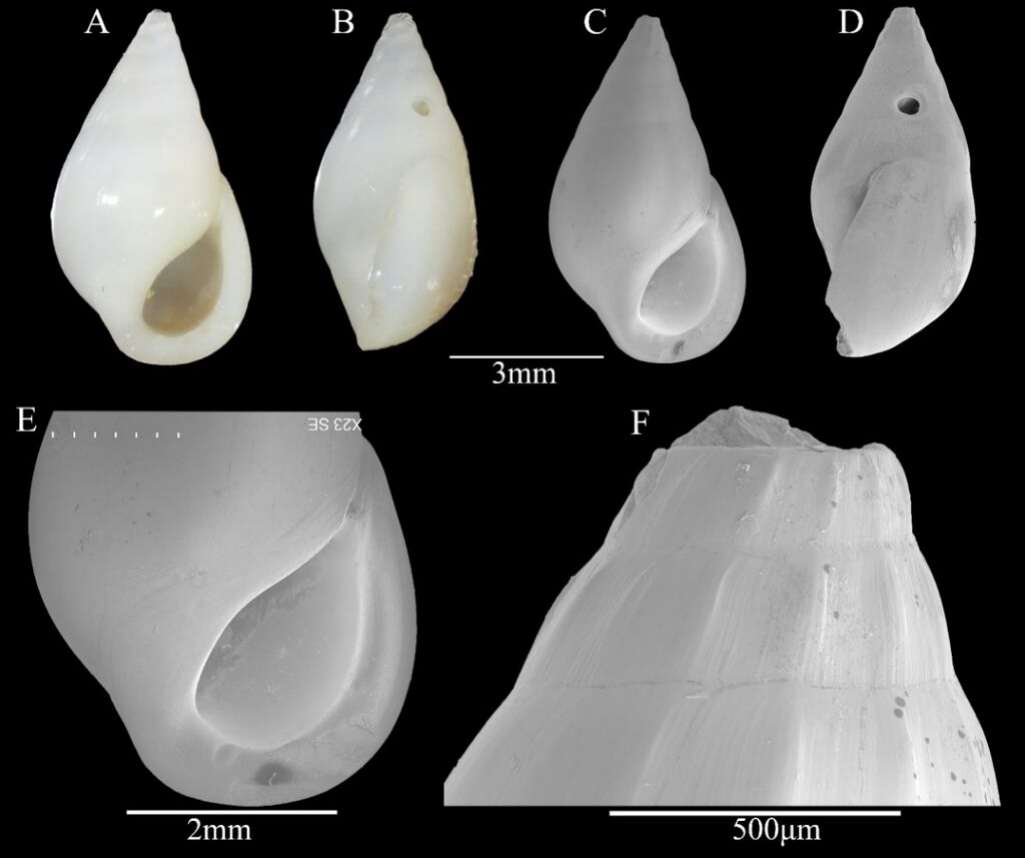
*Zebina tridentata* (Michaud, 1830). **A, B** shell illustration in colour; **C** scanning electron micrographs of frontal view of shell; **D** scanning electron micrographs of lateral view of shell; **E** detail of aperture; **F** scanning electron micrographs of lateral view of protoconch.

Shell minute (6.2 ± 0.02 mm in height, 3.8 ± 0.11 mm in width), thick-walled, solid, opaque-white, smooth and glossy, ovate-conical (Fig. 3A and B). Whorls approximately 7 (protoconch missing); body whorl inflated, its height exceeding that of spire; teleoconch whorls 1-3 with axial ribs, whorls 4 to body whorl smooth (Fig. 3C, D and F). Aperture elliptical, slightly prosocline, anteriorly rounded and posteriorly angulate; outer lip lacking external varix, but internally thickened, with a single small denticle on anterior interior edge; inner lip smooth; peristome with fine axial striations (Fig. 3E). Sutures indistinct.

#### Diagnosis

Shell conical，white, smooth and glossy; aperture ovate, oblique and somewhat channelled; outer lip swollen, with three teeth on the inner side; columella with a distinct callus at the upper part (Michaud 1830).

#### Distribution

Tropical and subtropical Indo-West Pacific (Hainan and Taiwan, China; Kyushu, Japan; Philippines), ranging from the Red Sea and Mozambique to Hawaii and Tuamotu Archipelago (Sleurs and Strack 2021).

#### Taxon discussion

Species of *Zebina* resemble those of the family Eulimidae, but are distinguished by distinct apertural features, typically exhibiting a robust outer lip varix and posterior channel. Additionally, *Zebina* shells tend to be thicker and more opaque. The diagnostic character of *Z. tridentata* is the presence of three denticles on the anterior interior edge of the outer lip (Michaud 1830). However, Cernohorsky (1978) noted that this species may exhibit 1–3 denticles. Therefore, we conclude that the denticle number in *Z. tridentata* exhibits intraspecific variation. In this study, the examined specimen possesses only a single denticle (Fig. 3E), yet consistent with other diagnostic features, we identify it as *Z. tridentata*.

### Stosicia annulata

(Dunker, 1860)

7A0086E7-055D-59DC-8A79-394C4CDD3A98

#### Materials

- **Type status:** Other material. **Occurrence:** catalogNumber: EM19; recordedBy: Qi Lu; individualCount: 2; lifeStage: adult; occurrenceID: FADFC8CD-7185-5D00-AA9F-69CEF492FF14; **Taxon:** taxonID: Native; scientificName: *Stosicia annulata*; originalNameUsage: *Alvania ligata* A. Gould, 1861, *Ravadia trochlearis* (A. Gould, 1861), Rissoina (Iravadia) annulata Dunker, 1860, *Rissoina annulata* Dunker, 1860, *Rissoina trochlearis* A. Gould, 1861, *Rissoina trochlearis* var. minor G. Nevill, 1885; **Location:** country: China; stateProvince: Hainan; locality: Danzhou; **Identification:** identifiedBy: Qi Lu; dateIdentified: 2023

#### Description

**Figure 4.**
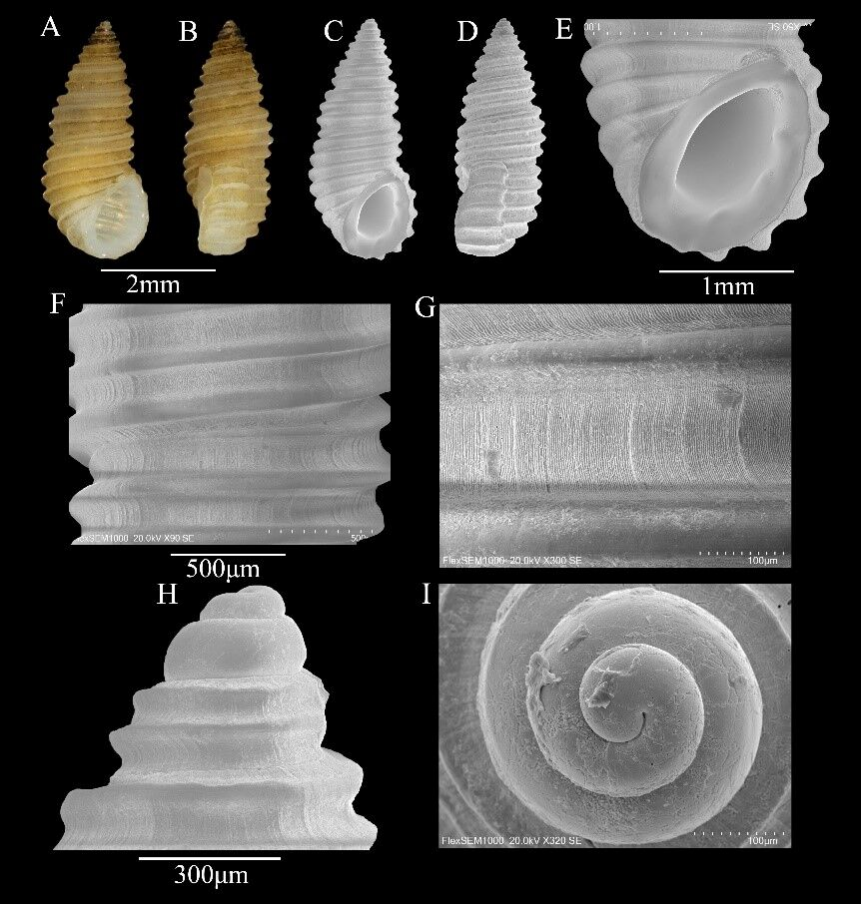
*Stosicia annulata* (Dunker, 1860). **A, B** shell illustration in colour; **C** scanning electron micrographs of frontal view of shell; **D** scanning electron micrographs of lateral view of shell; **E** detail of aperture; **F, G** detail of teleoconch surface; **H** scanning electron micrographs of lateral view of protoconch; **I** scanning electron micrographs of apical view of protoconch.

Shell minute (4 ± 0.01 mm in height, 1.9 ± 0.03 mm in width), thick-walled, white, but frequently stained pale reddish-brown by iron deposits (Fig. 4A and B). Whorls approximately 7, body whorl height greater than spire; teleoconch sculptured exclusively by strong spiral cords with axial lamellae between cords, interspaces about twice the width of cord (Fig. 4G); body whorl bearing 4 spiral cords (Fig. 4C and D). Sutures indistinct, shallow (Fig. 4F). Base with 3 spiral cords. Aperture elliptical, anteriorly canaliculate, posteriorly angulate; outer lip with strong external varix bearing spiral cords, interior smooth, but with 3 weakly-developed tubercular ridges; inner lip smooth to anterior canal, slightly arcuate, broad; peristome thickened (Fig. 4E). Protoconch multispiral (approximately 2.5 whorls), distinctly demarcated from teleoconch; clear boundary between first and second protoconch whorls, smooth (Fig. 4H and I).

#### Diagnosis

A species distinguished by its transverse ribs, of which seven are present on the last whorl (Dunker 1860).

#### Distribution

Widely distributed, currently known from the Bōsō and Noto Peninsulas to Kyushu (Japan), Southeast Asia, India, East Africa and Hainan Island (China) (Gajera et al. 2024).

#### Taxon discussion

The genus *Stosicia* Brusina, 1871 was divided by Ponder (1985) into two subgenera: *Stosicia* s.s. (with only spiral cords and multispiral protoconch) and Isseliella (with cancellate sculpture formed by axial and spiral ribs and paucispiral protoconch). These two subgenera have now been elevated to separate genera. *S. annulata* is reported from China for the first time and is, therefore, identified as a new record for the country.

## Discussion

Research on micromolluscs in China has historically been limited and taxonomic investigations focusing on the family Zebinidae are virtually absent. To date, records of zebinoids exist only from Taiwan (Liu 2008), while no species have previously been reported from other Chinese waters. The discovery and description of four Zebinidae species from Hainan Island, therefore, constitute a significant addition to the documentation of China’s molluscan diversity. These findings not only bridge a critical gap in the taxonomy of Zebinidae in the South China Sea, but also provide valuable baseline data for future investigations of tropical island reef ecosystems. It should be emphasised, however, that only four species were identified in this study and the available material was limited in number, with most specimens represented solely by empty shells. Such limitations are likely attributable to the restricted sampling frequency and spatial coverage of the surveyed habitats. Broader and more intensive sampling efforts, encompassing a wider range of sites, are expected to yield a greater number of specimens and potentially additional species of Zebinidae. The four species documented from Hainan Island exhibit clear morphological distinctions, corroborating the diagnostic characters traditionally employed in Zebinidae taxonomy, including shell sculpture, aperture morphology and columellar features. Nevertheless, it must be recognised that these morphological traits may be subject to variation influenced by environmental factors. Consequently, future research integrating molecular approaches will be essential to resolve phylogenetic relationships and to assess population-level differentiation within the group. In summary, our current understanding of Zebinidae in Chinese waters remains rudimentary. Continued, systematic surveys combined with integrative taxonomic approaches will not only refine the classification framework of micromolluscs in China, but also contribute to a more comprehensive understanding of tropical marine biodiversity in the region.

## Supplementary Material

XML Treatment for Schwartziella triticea

XML Treatment for Pandalosia subfirmata

XML Treatment for Zebina tridentata

XML Treatment for Stosicia annulata
